# Enhanced Efficiencies of Perovskite Solar Cells by Incorporating Silver Nanowires into the Hole Transport Layer

**DOI:** 10.3390/mi10100682

**Published:** 2019-10-10

**Authors:** Chien-Jui Cheng, Rathinam Balamurugan, Bo-Tau Liu

**Affiliations:** 1 Department of Chemical and Materials Engineering, National Yunlin University of Science and Technology, Yunlin 64002, Taiwan; 2 College of Future, National Yunlin University of Science and Technology, Yunlin 64002, Taiwan

**Keywords:** silver nanowire, inverted perovskite solar cell, hole transport layer, PEDOT:PSS

## Abstract

In this study, we incorporated silver nanowires (AgNWs) into poly(3,4-ethylenedioxythiophene):poly(styrene sulfonate) (PEDOT:PSS) as a hole transport layer (HTL) for inverted perovskite solar cells (PVSCs). The effect of AgNW incorporation on the perovskite crystallization, charge transfer, and power conversion efficiency (PCE) of PVSCs were analyzed and discussed. Compared with neat PEDOT:PSS HTL, incorporation of few AgNWs into PEDOT:PSS can significantly enhance the PCE by 25%. However, the AgNW incorporation may result in performance overestimation due to the lateral charge transfer. The corrosion of AgNWs with a perovskite layer was discussed. Too much AgNW incorporation may lead to defects on the interface between the HTL and the perovskite layer. An extra PEDOT:PSS layer over the pristine PEDOT:PSS-AgNW layer can prevent AgNWs from corrosion by iodide ions.

## 1. Introduction

Perovskite solar cells (PVSCs) are one of the most promising photovoltaic technologies due to their rapid increase in power conversion efficiency (PCE) from 3.8% to 24.2% in a very short period of time as well as offering specific advantages such as lightweight, low cost, and simple fabrication compared to traditional inorganic solar cells [1,2,3,4,5,6,7,8,9]. Among the PVSCs, inverted-type (planar) cells with a device structure of indium tin oxide (ITO)/hole transport layer (HTL)/perovskite/electron transport layer (ETL)/metal have emerged as an alternative to conventional PVSCs because of their low-temperature processability [10,11,12,13]. Inverted architecture devices can be processed at relatively low temperatures (~120 °C) compatible with flexible substrates. Moreover, inverted PVSCs show potential to be manufactured at a low cost and at an industrial scale. Poly(3,4-ethylenedioxythiophene):poly(styrene sulfonate) (PEDOT:PSS) is commonly used for the HTL of inverted PVSCs due to (a) high transparency in the visible range, (b) easy processing, (c) high work function, (d) tunable conductivity (10^−4^–10^−3^ S/cm), and (f) significant mechanical flexibility [14,15,16,17]. However, PEDOT:PSS also possesses some drawbacks, such as inefficient electron-blocking properties and limited chemical stability arising from its acidity and highly hygroscopic nature [1,18,19,20]. Several new materials, including NiO_x_ [21,22], CuSCN [23], PbS [24], and graphene oxide [25], have been investigated widely as potential alternatives to PEDOT:PSS to improve the resistance to electron transport and electrical conductivity. Besides, doping polyethylene oxide [26], MoO_x_ [27,28], GeO_2_ [29], or graphene oxide [30] into PEDOT:PSS can increase electrical conductivity and hole extraction, reduce the series resistance, and thereby enhance PCE. It was also found that adjusting the pH value of PEDOT:PSS by NaOH [31], KOH [32], or dopamine [33] could promote effectively the cell stability. Although numerous studies have reported improvement or alternatives for PEDOT:PSS, it still remains a challenge to achieve high efficiency.

Compared with 0-D metal nanoparticles, 1-D silver nanowires (AgNWs) display superior electrical conductivity due to the fast electron transport on their axial direction. Applying AgNWs in transparent conductive electrodes show not only excellently flexible properties but also a very high ratio of DC conductivity to optical conductivity, indicating AgNWs are one of the best potential replacements for indium tin oxides [34]. AgNW networks have been improved extremely in terms of the electrical conductivity, optical properties, and manufacturing [35,36,37,38,39]. Therefore, some studies have used AgNWs to fabricate the top electrodes [40,41,42] and bottom window electrodes [43,44] of PVSCs, revealing feasibility of AgNW electrodes on PVSCs. In this study, we incorporated AgNWs into the PEDOT:PSS as a HTL for inverted PVSCs. The effect of AgNWs on the formation of the perovskite layer, charge transfer resistance, and PCE of PVSCs were studied with respect to varying the concentration of AgNWs. Compared with neat PEDOT:PSS HTL, incorporation of few AgNWs into PEDOT:PSS significantly enhances the PCE by one-fourth. The corrosion of AgNWs with a perovskite layer was discussed.

## 2. Experimental Setup

### 2.1. Materials 

ITO-coated glass substrates (7 Ω·sq^−1^) were purchased from Ruilong optoelectronics (Taiwan). PDOT:PSS and 6,6-phenyl-C61-butyric acid methyl ester (PCBM) were purchased from Grand-Hand (Taiwan). Methylamine and silver nitrate were purchased from Showa Chemical (Tokyo, Japan). Hydrogen iodide and lead (II) iodide were purchased from Alfa Aesar (Shanghai, China) and Acros Organics (Geel, Belgium), respectively. Hexadecyl trimethyl ammonium bromide (CTAB), anhydrous dimethyl sulfoxide (DMSO), and polyvinylpyrrolidone (PVP) were purchased from Sigma-Aldrich (Saint Louis, MO, USA) and used as received. Methylamine iodide (MAI) was synthesized from methylamine and hydroiodic acid (see supporting information). Other chemicals and solvents such as chloroform, dimethyl formamide (DMF) were purchased from J.T. Baker (Phillipsburg, NJ, USA) and used without further purification. 

### 2.2. Device Fabrication

Typically, the ITO-coated glass substrate was masked by PI tape in a T shape (2 × 1.5 cm) and then the unmasked portion of the ITO was etched with zinc powder and diluted HCl (<6 M). After few seconds, the residual zinc powder was removed by washed with ethanol. Then the mask was removed and cleaned three times with detergent, ethanol, and acetone. AgNWs were synthesized as reported previously [45,46], with some of the parameters modified slightly to obtain fine AgNWs (see supporting information). The mixture of PEDOT:PSS and AgNWs EG solution (0, 0.17, 0.51, and 0.85 wt%) in the weight ratio of 20:1 was coated on ITO glass at 3000 rpm for 60 s and then dried by heating at 120 °C for 10 min. MAI and PbI_2_ were mixed in DMSO/DMF in the volumetric ratio of 4:6 at 60 °C to obtain a 1.2-M MAPbI_3_ solution. The solution was coated on the HTL under the conditions of 1000 rpm for 10 s, followed by 5000 rpm for 20 s and heating at 100 °C to complete the film formation. 10 mg of PCBM and 0.8 mg of CTAB were dissolved in 500 μL of chloroform with ultrasonication and then spin-coated onto the perovskite layer at 1250 rpm for 30 s. Finally, near 100-nm thick silver was thermally deposited on the ETL layer by a thermal evaporator (Kao-Duen Tech, Taiwan) under a vacuum of 5 × 10^−6^ torr and a plating rate of 0.8–0.9 Å/s. The structure of the PVSC is shown schematically in Figure S1.

### 2.3. Measurements and Characterization

The crystalline phase of AgNWs and MAPbI_3_ were characterized through X-ray diffraction (XRD) using an X-ray diffractometer (Miniflex II, Rigaku, Tokyo, Japan) and Cu Kα radiation (wavelength 0.15418 nm) with a fixed operating voltage of 30 kV and a fixed current of 15 mA. The sheet resistances of the PEDOT:PSS incorporated with AgNWs were measured using a four-pin probe meter (Loresta-GP, Mitsubishi Chemical, Tokyo, Japan) with an MCP-T610 probe. The morphology of AgNWs, MAPbI_3_, and the devices were examined using a field-emission scanning electron microscope (SEM) (JSM-7401F, JEOL, Tokyo, Japan). The length of AgNWs was determined using an optical microscope (OM) (M835, M&T Optics, Taiwan). The absorption and emission spectra of the ITO/PEDOT:PSS-AgNW/MAPbI_3_ were determined using a UV-Vis spectrometer (V770, Jasco, Tokyo, Japan) and a fluorescence spectrometer (LS-55, Perkin Elmer, Waltham, MA, USA), respectively. The photocurrent density-voltage (J-V) characteristics were measured under irradiation of 100 mW·cm^-2^ using a solar simulator (MFS-PV, Hong-Ming Technology, Taiwan) equipped with a source meter (Keithley 2400, Keithley Instruments, Cleveland, OH, USA). Electrochemical impedance spectra (EIS) were measured over the frequency range of 50 mHz–100 kHz with a potential perturbation of 10 mV using an electrochemical workstation (Zennium, Zahner Elektrik, Kronach, Germany). 

## 3. Results and Discussion

**Figure 1:**
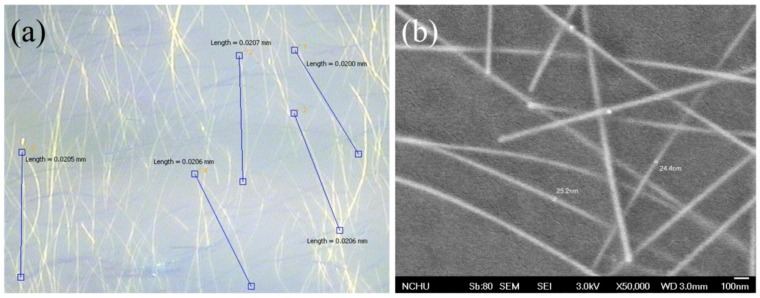
Typical (**a**) optical microscope (OM) and (**b**) scanning electron microscope (SEM) images of as-prepared AgNWs.

During the AgNW synthesis process, both length and diameter of AgNWs increase with reaction time. In order to prevent the AgNWs from exceeding HTL in thickness to conduct charge recombination or current leakage, we shortened the reaction time to make finer ones in our experiments. The as-prepared AgNWs had lengths of ~20 μm and diameters of 20–30 nm (Figure 1), confined by the thickness of the HTL. Although PEDOT:PSS is electrically conductive in nature, the sheet resistance of the PEDOT:PSS layer really decreases with the increase of AgNW content (Figure S2), revealing the AgNW incorporation can improve the conductivity of the HTL. 

**Figure 2:**
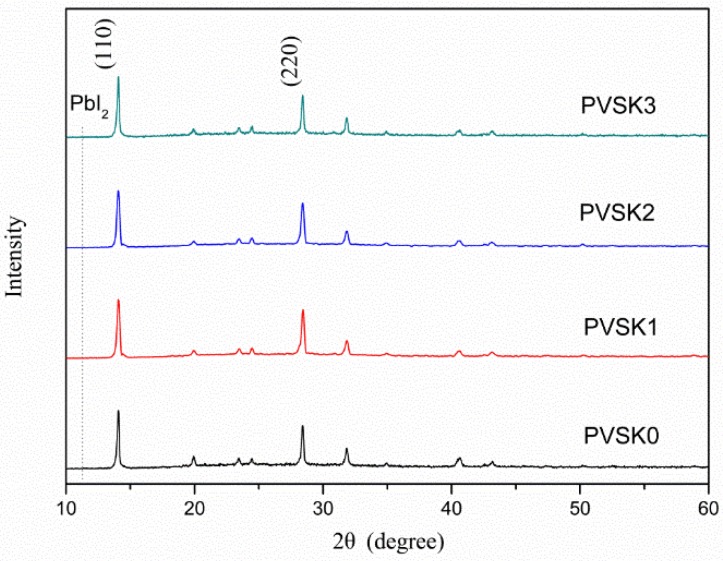
X-ray diffraction (XRD) patterns of the MAPbI_3_ layers with various AgNW contents.

**Figure 3:**
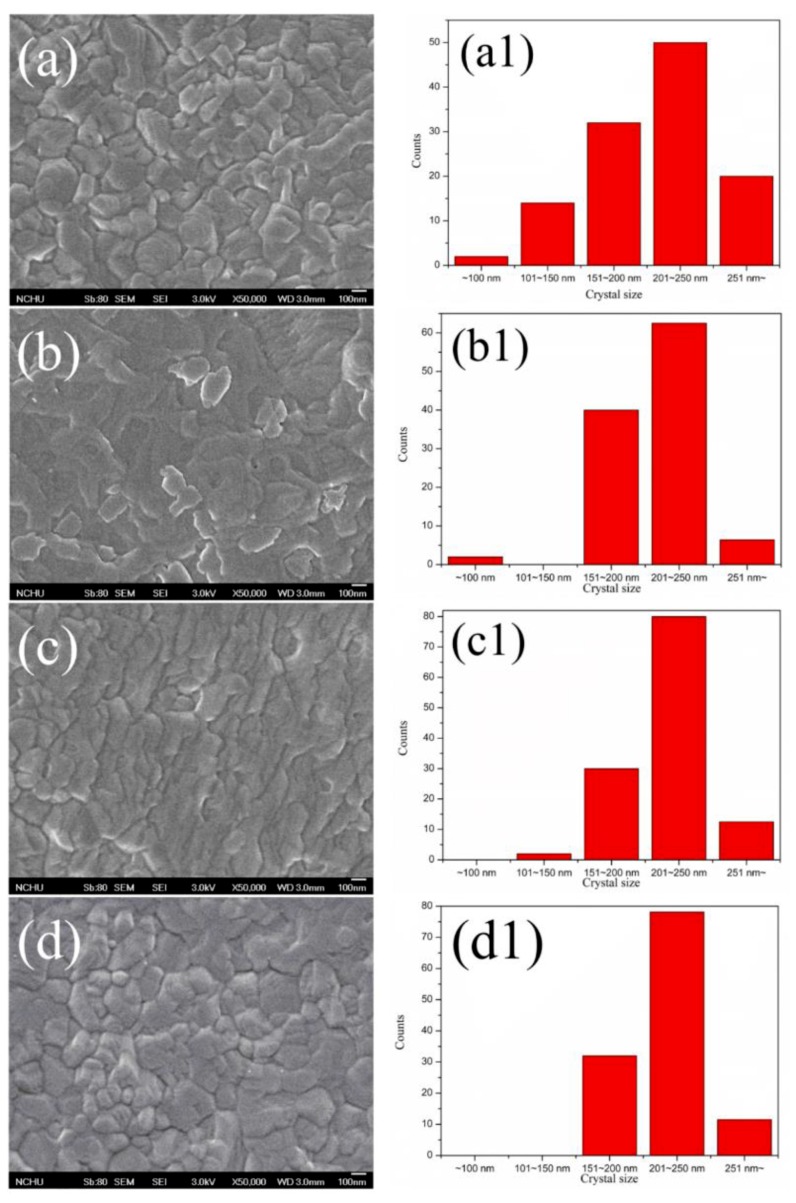
Scanning electron microscopy (SEM) images and crystal size distributions of the MAPbI_3_ layers with various AgNW contents. (**a**,**a1**) 0 wt%, (**b**,**b1**) 0.17 wt%, (**c**,**c1**) 0.51 wt%, (**d**,**d1**) 0.85 wt%.

**Figure 4:**
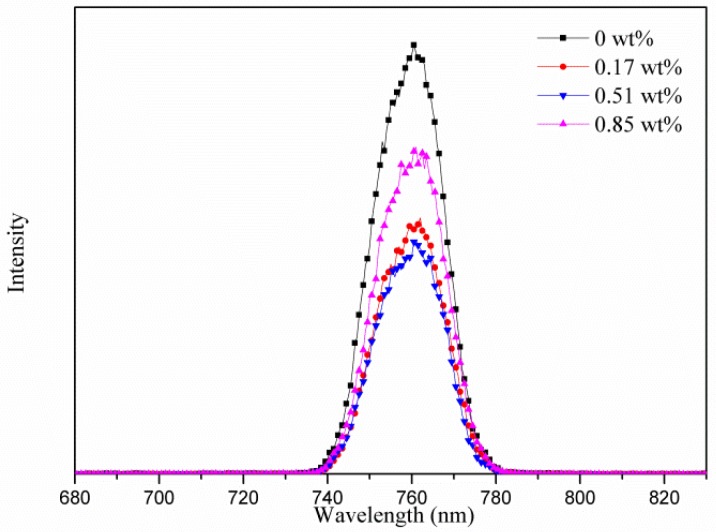
PL spectra of the MAPbI_3_ layers with various AgNW contents.

**Figure 5:**
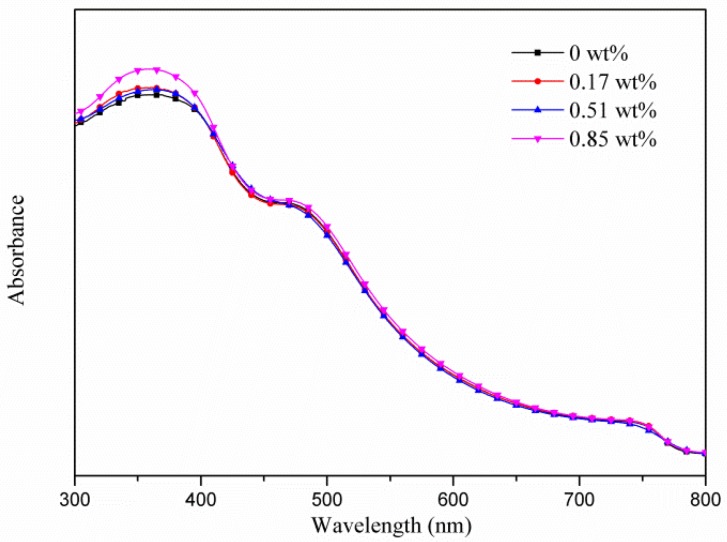
UV-Vis spectra of the MAPbI_3_ layers with various AgNW contents.

Observing in the XRD patterns (Figure 2), neither an obvious change on the crystallinity of MAPbI_3_ for the PEDOT:PSS-AgNW HTL nor a peak attributed to PbI_2_ (12.7°) was found, implying that AgNW incorporation did not lead to PbI_2_ separated out from MAPbI_3_. Note that the XRD patterns did not display the main peak at 37.8° corresponding with the (111) plane of AgNWs because the AgNWs are few and sheltered by the MAPbI_3_ layer. Figure 3 shows the top-view SEM images of the morphology of the MAPbI_3_ layer upon the HTL with various AgNW contents. The average crystal size of MAPbI_3_ in the range of 200–220 nm is not strongly dependent on the AgNW contents in the HTL. However, the PL intensity of MAPbI_3_ decreases with increasing AgNW amount in the HTL but increases with further increasing AgNW amount (Figure 4). The fluorescence intensity results from the electron-hole recombination. Therefore, the PL analysis implies that moderate AgNW incorporation can reduce charge recombination rate. The AgNW incorporation also induces the slight increase on the absorption in the range of 300–400 nm (Figure 5), which may arise from surface plasmon resonance absorption of AgNWs [47]. 

**Figure 6:**
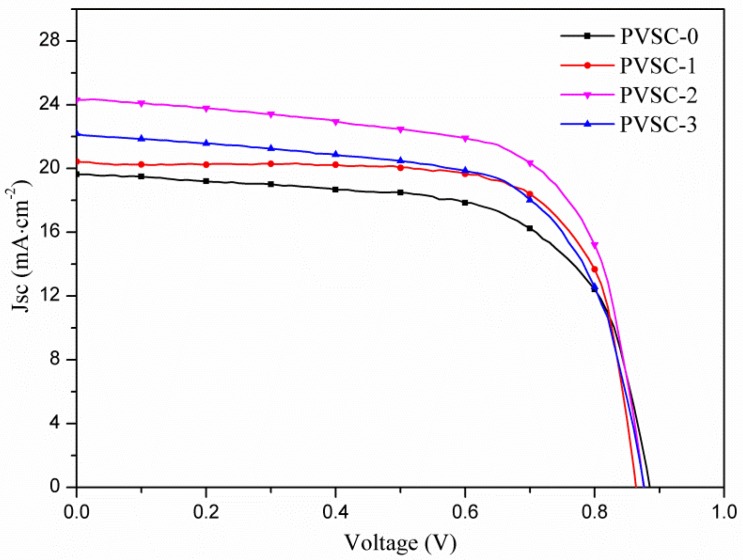
Photocurrent density-voltage curves of perovskite solar cells (PVSCs) with various AgNW incorporations.

**Figure 7:**
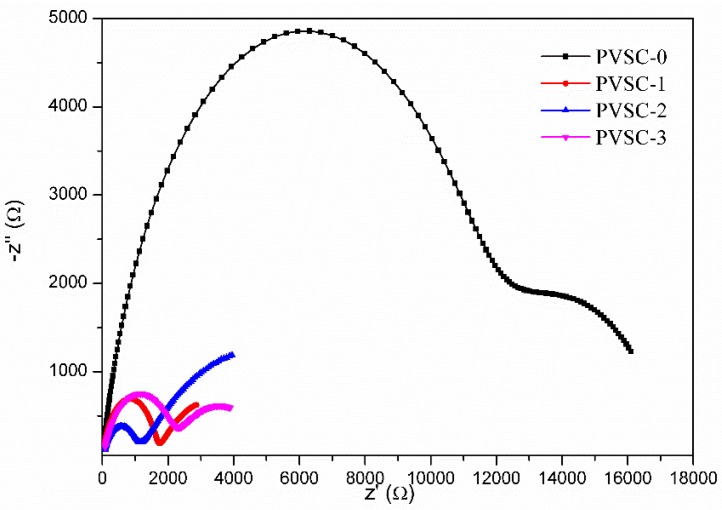
Nyquist plots of the electrochemical impedance spectra of PVSCs with various AgNW incorporations.

Figure 6 shows the J-V curves of the PVSCs with various AgNW incorporations in HTL. The corresponding characteristic properties are summarized in Table 1. The experimental results show that the PCEs of the PVSCs increase with increasing AgNW content in the HTL, reached a maximum at PVSC-2 (0.51 wt% AgNWs), and decreased with further increasing AgNW content (PVSC-3). The order is reciprocal to the PL intensity. Because the decrease of the fluorescence intensity (quenching) indicates the decrease of electron-hole recombination rate, the enhancement on PCE caused by the AgNW incorporation may result from the rapid hole transfer away through AgNWs to suppress the charge recombination. The PCE can be enhanced by approximately 25% compared to the pristine cell (PVSC-0). Figure 7 displays the Nyquist plots of the EIS for the PVSCs with various AgNW incorporations in the HTL. As listed in Table 1, the external (*R*_1_), interface (*R*_2_), and charge recombination resistances (*R*_3_) appears sequentially from high to low frequency, respectively. With the increase of AgNW content, *R*_2_ decreases significantly, whereas *R*_3_ reaches a maximum at 0.51 wt% AgNW incorporation. The results indicate that the AgNW incorporation decreases the interface resistance and the rate of electron-hole recombination, which is consistent with PL and J-V analyses. However, too much AgNW incorporation (0.85 wt% AgNWs) leads to high PL intensity, low PCE, high interface resistance, and low resistance of charge combination. Figure S3 shows the monochromatic incident photon-to-electron conversion efficiency (IPCE) spectrum of PVSC-2 (the best cell). The calculated *J*_SC_ from integration of the IPCE over the AM 1.5 G is 20.6 mA/cm^2^, which is smaller than the value of 24.3 mA/cm^2^ obtained from J-V curve listed in Table 1. The discrepancy in the two methods may arise from different light intensity, mismatch in the absorption spectrum, or degradation of device [48,49]. However, the difference in *J*_SC_ values obtained from the IPCE and J-V curve is much larger than that reported in literature. To realize the cause of the discrepancy, we measured the performance of the solar cells with a mask. The *J*_SC_ and PCE led to a 5–10% reduction after applying a mask. Therefore, the enhancement on *J*_SC_ and PCE by incorporating AgNWs may result partially from the lateral charge transport, which overestimates the performance.

**Figure 8:**
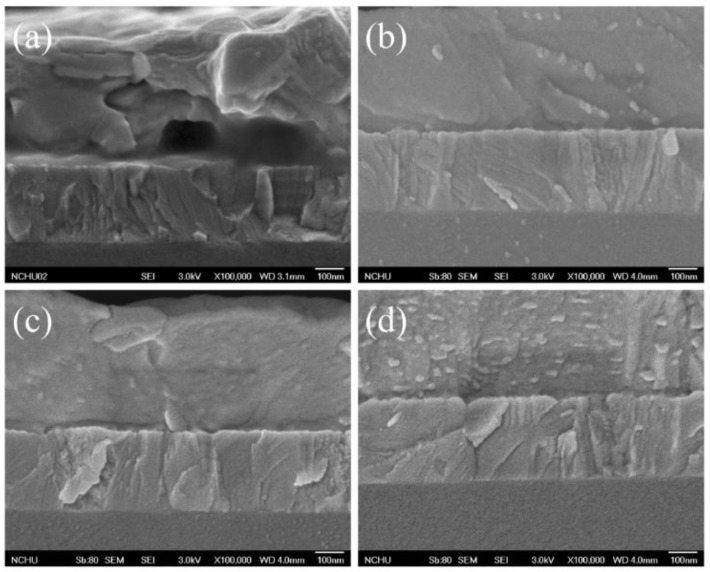
Cross-sectional SEM images of (**a**) PVSC-3, (**b**) PVSC-3-1L, (**c**) PVSC-3-2L, (**d**) PVSC-3-3L.

**Figure 9:**
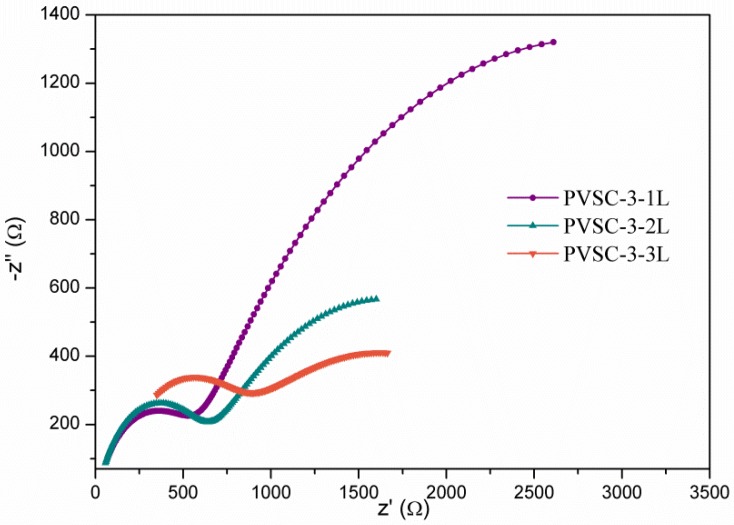
Nyquist plots of the electrochemical impedance spectra of PVSC-3 with coating extra PEDOT:PSS layers.

The SEM image reveals that some defects were found in the HTL for high AgNW incorporation (Figure 8a). We infer that too many AgNWs might have protrude out of the HTL and contacted the MAPbI_3_ solution when the subsequent perovskite layer was coated. The iodide ion in the MAPbI_3_ might react with AgNWs to form defects [50]. A protection layer may prevent AgNWs from reacting with halogen ions to form nonconducting silver halide, deteriorating the optoelectronic properties [51,52]. In the previous study [45], we proved that PEDOT:PSS can prevent AgNWs from corrosion by the iodine electrolyte. In order to verify the argument, we spin-coated extra PEDOT:PSS layers upon the pristine PEDOT:PSS-AgNW layer to prevent AgNWs from touching the MAPbI_3_ solution. After coating an extra one, two, or three PEDOT:PSS layers, no defects were found in the interface between the MAPbI_3_ layer and the PEDOT:PSS–AgNW layer (Figure 8b–d). The PCE was increased from 12.14% to 13.04% with one extra PEDOT:PSS layer, whereas too much PEDOT:PSS (spin-coating PEDOT:PSS two or three times) makes the PCE decline (Figure S4 and Table 2). Figure 9 shows the Nyquist plots of the EIS for the PVSCs with extra PEDOT:PSS layers. The resistance of charge recombination (*R*_3_) increases with one covering PEDOT:PSS layer (PVSC-3-1L) to prevent against iodine-mediated corrosion. However, too much PEDOT:PSS coating decreases the resistance of charge recombination, which increases the probability of electron-hole recombination due to the increase of distance of charge transport and thereby decreases the PCE.

## 4. Conclusions

We prepared the inverted PVSCs with incorporating AgNWs into PEDOT:PSS as a HTL. The AgNW incorporation increased the rate of charge transport and suppressed the electron-hole recombination. Incorporation of 0.51 wt% AgNWs revealed a maximum improvement, being a 25% enhancement on the PCE. However, the AgNW incorporation may overestimate performance due to the lateral charge transfer. Too much AgNW incorporation may result in the contact of AgNWs with the MAPbI_3_ solution, thereby forming defects on the interface between the HTL and the MAPbI_3_ layer. An extra PEDOT:PSS layer over the pristine PEDOT:PSS-AgNW layer can prevent AgNWs from corrosion by iodide ions, whereas too much PEDOT:PSS protection may increase the distance of charge transport and may thus decrease the PCE.

## Figures and Tables

**Table 1:** Photovoltaic characteristics of PVSCs incorporated with various AgNW amounts in the hole transport layer.

Samples	AgNW Solution (wt%)	Voc (V)	*J*sc (mA·cm^−2^)	FF (%)	*η* (%)	*R*_1_ (Ω)	*R*_2_ (kΩ)	*R*_3_ (kΩ)
PVSC-0	0	0.89	19.6	65.4	11.4	7.6	11.6	5.8
PVSC-1	0.17	0.86	20.4	73.0	12.9	5.9	1.6	3.3
PVSC-2	0.51	0.88	24.3	67.0	14.3	10.2	1.0	7.4
PVSC-3	0.85	0.84	22.2	64.9	12.1	10.9	2.1	3.0

**Table 2:** Photovoltaic characteristics of PVSC-3 with coating extra PEDOT:PSS layers over the pristine AgNW-PEDOT:PSS hole transport layer.

Samples	AgNW Solution (wt%)	Number of Extra PEDOT:PSS Layers	Voc (V)	*J*sc (mA·cm^-2^)	FF (%)	*η* (%)	*R*_1_ (Ω)	*R*_2_ (Ω)	*R*_3_ (kΩ)
PVSC-3-1L	0.85	1	0.97	21.8	51.5	13.0	18.1	514.8	4.6
PVSC-3-2L	0.85	2	0.93	19.1	51.4	9.1	20.4	567.9	2.1
PVSC-3-3L	0.85	3	0.92	19.4	54.5	9.8	113.7	562.5	1.9

## Supplementary Materials

The following are available online at https://www.mdpi.com/2072-666X/10/10/682/s1, Figure S1: Schematic representation of structure of the cell device; Figure S2: Variation on the sheet resistance of HTL with various AgNW contents; Figure S3: IPCE spectrum of PVSC-2; Figure S4: Photocurrent density-voltage curves of PVSCs with coating extra PEDOT:PSS layers.

## References

[1] Neophytou M., Griffiths J., Fraser J., Kirkus M., Chen H., Nielsen C.B., McCulloch I. (2017). High mobility, hole transport materials for highly efficient PEDOT:PSS replacement in inverted perovskite solar cells. J. Mater. Chem. C.

[2] Xu C., Liu Z., Lee E.-C. (2018). High-performance metal oxide-free inverted perovskite solar cells using poly(bis(4-phenyl)(2,4,6-trimethylphenyl)amine) as the hole transport layer. J. Mater. Chem. C.

[3] Grätzel M. (2014). The light and shade of perovskite solar cells. Nat. Mater..

[4] Stranks S.D., Snaith H.J. (2015). Metal-halide perovskites for photovoltaic and light-emitting devices. Nat. Nanotech..

[5] Liu T., Chen K., Hu Q., Zhu R., Gong Q. (2016). Inverted perovskite solar cells: Progresses and perspectives. Adv. Energy Mater..

[6] Meng L., You J., Guo T.-F., Yang Y. (2016). Recent advances in the inverted planar structure of perovskite solar cells. Acc. Chem. Res..

[7] Kojima A., Teshima K., Shirai Y., Miyasaka T. (2009). Organometal halide perovskites as visible-light sensitizers for photovoltaic cells. J. Am. Chem. Soc..

[8] Sun K., Li P., Xia Y., Chang J., Ouyang J. (2015). Transparent conductive oxide-free perovskite solar cells with PEDOT:PSS as transparent electrode. ACS Appl. Mater. Interfaces.

[9] Li P.-S., Balamurugan R., Liu B.-T., Lee R.-H., Chou H.-T. (2019). MAPbI_3_ incorporated with carboxyl group chelated titania for planar perovskite solar cells in low-temperature process. Nanomaterials.

[10] Shahbazi S., Tajabadi F., Shiu H.S., Sedighi R., Jokar E., Gholipour S., Taghavinia N., Afshar S., Diau E.W.G. (2016). An easy method to modify PEDOT:PSS/perovskite interfaces for solar cells with efficiency exceeding 15%. RSC Adv..

[11] You J., Hong Z., Yang Y., Chen Q., Cai M., Song T.-B., Chen C.-C., Lu S., Liu Y., Zhou H. (2014). Low-temperature solution-processed perovskite solar cells with high efficiency and flexibility. ACS Nano.

[12] Malinkiewicz O., Yella A., Lee Y.H., Espallargas G.M., Graetzel M., Nazeeruddin M.K., Bolink H.J. (2013). Perovskite solar cells employing organic charge-transport layers. Nat. Photon..

[13] Docampo P., Ball J.M., Darwich M., Eperon G.E., Snaith H.J. (2013). Efficient organometal trihalide perovskite planar-heterojunction solar cells on flexible polymer substrates. Nat. Commun..

[14] Thomas J.P., Zhao L., McGillivray D., Leung K.T. (2014). High-efficiency hybrid solar cells by nanostructural modification in PEDOT:PSS with co-solvent addition. J. Mater. Chem. A.

[15] Lin Y.-J., Ni W.-S., Lee J.-Y. (2015). Effect of incorporation of ethylene glycol into PEDOT:PSS on electron phonon coupling and conductivity. J. Appl. Phys..

[16] Lin Y.-J., Chin Y.-M., Wu C.-Y., Liu D.-S. (2014). Electron-phonon coupling modification and carrier mobility enhancement in poly(3,4-ethylenedioxythiophene) doped with poly(4-styrenesulfonate) films by ultraviolet irradiation. J. Appl. Phys..

[17] Lin J.-H., Zeng J.-J., Su Y.-C., Lin Y.-J. (2012). Current transport mechanism of heterojunction diodes based on the reduced graphene oxide-based polymer composite and n-type Si. Appl. Phys. Lett..

[18] Xiao Z., Bi C., Shao Y., Dong Q., Wang Q., Yuan Y., Wang C., Gao Y., Huang J. (2014). Efficient, high yield perovskite photovoltaic devices grown by interdiffusion of solution-processed precursor stacking layers. Energy Environ. Sci..

[19] Li H., Fu K., Boix P.P., Wong L.H., Hagfeldt A., Grätzel M., Mhaisalkar S.G., Grimsdale A.C. (2014). Hole-transporting small molecules based on thiophene cores for high efficiency perovskite solar cells. ChemSusChem.

[20] Labban A.E., Chen H., Kirkus M., Barbe J., Del Gobbo S., Neophytou M., McCulloch I., Eid J. (2016). Improved efficiency in inverted perovskite solar cells employing a novel diarylamino-substituted molecule as PEDOT:PSS replacement. Adv. Energy Mater..

[21] You J., Meng L., Song T.-B., Guo T.-F., Yang Y., Chang W.-H., Hong Z., Chen H., Zhou H., Chen Q. (2015). Improved air stability of perovskite solar cells via solution-processed metal oxide transport layers. Nat. Nanotech..

[22] Jeng J.-Y., Chen K.-C., Chiang T.-Y., Lin P.-Y., Tsai T.-D., Chang Y.-C., Guo T.-F., Chen P., Wen T.-C., Hsu Y.-J. (2014). Nickel oxide electrode interlayer in ch3nh3pbi3 perovskite/PCBM planar-heterojunction hybrid solar cells. Adv. Mater..

[23] Ye S., Sun W., Li Y., Yan W., Peng H., Bian Z., Liu Z., Huang C. (2015). CuSCN-based inverted planar perovskite solar cell with an average PCE of 15.6%. Nano Lett..

[24] Hu L., Wang W., Liu H., Peng J., Cao H., Shao G., Xia Z., Ma W., Tang J. (2015). PbS colloidal quantum dots as an effective hole transporter for planar heterojunction perovskite solar cells. J. Mater. Chem. A.

[25] Wu Z., Bai S., Xiang J., Yuan Z., Yang Y., Cui W., Gao X., Liu Z., Jin Y., Sun B. (2014). Efficient planar heterojunction perovskite solar cells employing graphene oxide as hole conductor. Nanoscale.

[26] Huang X., Wang K., Yi C., Meng T., Gong X. (2016). Efficient perovskite hybrid solar cells by highly electrical conductive PEDOT:PSS hole transport layer. Adv. Energy Mater..

[27] Kim D.B., Yu J.C., Nam Y.S., Kim D.W., Jung E.D., Lee S.Y., Lee S., Park J.H., Lee A.-Y., Lee B.R. (2016). Improved performance of perovskite light-emitting diodes using a PEDOT:PSS and MoO_3_ composite layer. J. Mater. Chem. C.

[28] Jiang Y., Li C., Liu H., Qin R., Ma H. (2016). Poly(3,4-ethylenedioxythiophene):poly(styrenesulfonate) (PEDOT:PSS)–molybdenum oxide composite films as hole conductors for efficient planar perovskite solar cells. J. Mater. Chem. A.

[29] Wang Z.-K., Li M., Yuan D.-X., Shi X.-B., Ma H., Liao L.-S. (2015). Improved hole interfacial layer for planar perovskite solar cells with efficiency exceeding 15%. ACS Appl. Mater. Interfaces.

[30] Luo H., Lin X., Hou X., Pan L., Huang S., Chen X. (2017). Efficient and air-stable planar perovskite solar cells formed on graphene-oxide-modified PEDOT:PSS hole transport layer. Nano-Micro Lett..

[31] Tsai T.-C., Chang H.-C., Chen C.-H., Huang Y.-C., Whang W.-T. (2014). A facile dedoping approach for effectively tuning thermoelectricity and acidity of PEDOT:PSS films. Org. Electron..

[32] Wang Q., Chueh C.-C., Eslamian M., Jen A.K.Y. (2016). Modulation of PEDOT:PSS pH for efficient inverted perovskite solar cells with reduced potential loss and enhanced stability. ACS Appl. Mater. Interfaces.

[33] Huang J., Wang K.-X., Chang J.-J., Jiang Y.-Y., Xiao Q.-S., Li Y. (2017). Improving the efficiency and stability of inverted perovskite solar cells with dopamine-copolymerized PEDOT:PSS as a hole extraction layer. J. Mater. Chem. A.

[34] Liu B.-T., Huang S.-X. (2014). Transparent conductive silver nanowire electrodes with high resistance to oxidation and thermal shock. RSC Adv..

[35] Kim D., Ko Y., Kim W., Kim D., You J. (2017). Highly efficient silver nanowire/PEDPT:PSS composite microelectrodes via poly(ethylene glycol) photolithography. Opt. Mater. Express.

[36] Kim S., Kim S.Y., Kim J., Kim J.H. (2014). Highly reliable AgNW/PEDOT:PSS hybrid films: Efficient methods for enhancing transparency and lowering resistance and haziness. J. Mater. Chem. C.

[37] Liu Y., Feng J., Ou X.-L., Cui H., Xu M., Sun H.-B. (2016). Ultrasmooth, highly conductive and transparent PEDOT:PSS/silver nanowire composite electrode for flexible organic light-emitting devices. Org. Electron..

[38] Choi D.Y., Kang H.W., Sung H.J., Kim S.S. (2013). Annealing-free, flexible silver nanowire–polymer composite electrodes via a continuous two-step spray-coating method. Nanoscale.

[39] Kim S., Kim S.Y., Chung M.H., Kim J., Kim J.H. (2015). A one-step roll-to-roll process of stable AgNW/PEDOT:PSS solution using imidazole as a mild base for highly conductive and transparent films: Optimizations and mechanisms. J. Mater. Chem. C.

[40] Han K., Xie M., Zhang L., Yan L., Wei J., Ji G., Luo Q., Lin J., Hao Y., Ma C.-Q. (2018). Fully solution processed semi-transparent perovskite solar cells with spray-coated silver nanowires/ZnO composite top electrode. Sol. Energy Mater. Sol. Cells.

[41] Xie M., Lu H., Zhang L., Wang J., Luo Q., Lin J., Ba L., Liu H., Shen W., Shi L. (2018). Fully solution-processed semi-transparent perovskite solar cells with ink-jet printed silver nanowires top electrode. Sol. RRL.

[42] Fang Y., Wu Z., Li J., Jiang F., Zhang K., Zhang Y., Zhou Y., Zhou J., Hu B. (2018). High-performance hazy silver nanowire transparent electrodes through diameter tailoring for semitransparent photovoltaics. Adv. Funct. Mater..

[43] Jin Y., Sun Y., Wang K., Chen Y., Liang Z., Xu Y., Xiao F. (2018). Long-term stable silver nanowire transparent composite as bottom electrode for perovskite solar cells. Nano Res..

[44] Chen H., Li M., Wen X., Yang Y., He D., Choy W.C.H., Lu H. (2019). Enhanced silver nanowire composite window electrode protected by large size graphene oxide sheets for perovskite solar cells. Nanomaterials.

[45] Liu B.-T., Wang Z.-T. (2016). Graphene oxide/poly(3,4-ethylenedioxythiophene):polystyrenesulfonate layers on silver nanowire working electrodes enhance the power conversion efficiencies of dye-sensitized solar cells in a low temperature process. RSC Adv..

[46] Liu B.-T., Li C.-D. (2019). Highly conductive and fine lines of silver nanowires fabricated by evaporative self-assembly. J. Taiwan Inst. Chem. Eng..

[47] Sun Y., Yin Y., Mayers B.T., Herricks T., Xia Y. (2002). Uniform silver nanowires synthesis by reducing AgNO_3_ with ethylene glycol in the presence of seeds and poly(vinyl pyrrolidone). Chem. Mater..

[48] Liu T., Liu W., Zhu Y., Wang S., Wu G., Chen H. (2017). All solution processed perovskite solar cells with Ag@Au nanowires as top electrode. Sol. Energy Mater. Sol. Cells.

[49] Sutanto A.A., Lan S., Cheng C.-F., Mane S.B., Wu H.-P., Leonardus M., Xie M.-Y., Yeh S.-C., Tseng C.-W., Chen C.-T. (2017). Solvent-assisted crystallization via a delayed-annealing approach for highly efficient hybrid mesoscopic/planar perovskite solar cells. Sol. Energy Mater. Sol. Cells.

[50] Kang S., Jeong J., Cho S., Yoon Y.J., Park S., Lim S., Kim J.Y., Ko H. (2019). Ultrathin, lightweight and flexible perovskite solar cells with an excellent power-per-weight performance. J. Mater. Chem. A.

[51] Kim A., Lee H., Kwon H.-C., Jung H.S., Park N.-G., Jeong S., Moon J. (2016). Fully solution-processed transparent electrodes based on silver nanowire composites for perovskite solar cells. Nanoscale.

[52] Lee E., Ahn J., Kwon H.-C., Ma S., Kim K., Yun S., Moon J. (2018). All-solution-processed silver nanowire window electrode-based flexible perovskite solar cells enabled with amorphous metal oxide protection. Adv. Energy Mater..

