# Supplementary material for: Enhanced Efficiencies of Perovskite Solar Cells by Incorporating Silver Nanowires into the Hole Transport Layer
Source: Micromachines (Basel). 2019 Oct 10;10(10):682. doi: 10.3390/mi10100682 (PMC6843368; doi:10.3390/mi10100682)
Supplement: Supplementary file 1 [file micromachines-10-00682-s001.pdf]

Supporting Information

Enhanced efficiencies of perovskite solar cells by incorporating silver nanowires into the hole transport layer

Chien-Jui Cheng [1], Rathinam Balamurugan [2] and Bo-Tau Liu [1,*]

1 Department of Chemical and Materials Engineering, National Yunlin University of Science and Technology, Yunlin 64002, Taiwan
2 College of Future, National Yunlin University of Science and Technology, Yunlin 64002, Taiwan
* Correspondence: liubo@yuntech.edu.tw; Tel.: 886-5-534-2601

Synthesis of CH_3NH_3I

20 ml of hydroiodic acid was slowly dropped in to 50 ml of CH_3NH_2 and stirred for 2 h at 0 °C. Then the solvent was removed by rotary evaporator. The as-prepared powders was dissolved in ethanol and re-crystalized with diethyl ether. The process was repeated three times, resulting in CH_3NH_3I.

Preparation of AgNWs

0.2 M NaCl (80 μL), 1 M $AgNO_3$ (20 μL) , and 0.3 M polyvinylpyrrolidone (36 ml, MW: 40,000, Sigma-Aldrich) were mixed in a reaction vessel at 150 °C. 1 M $AgNO_3$ (4 ml) was then added slowly into the mixture. The solvent for all the above-mentioned solutions is ethylene glycol (EG). After the color of the mixture becomes silver-whitish, the resulting mixture was washed three times with EtOH through centrifugation, resulting in AgNWs. The as-prepared AgNWs were dispersed into EtOH as AgNW solution.

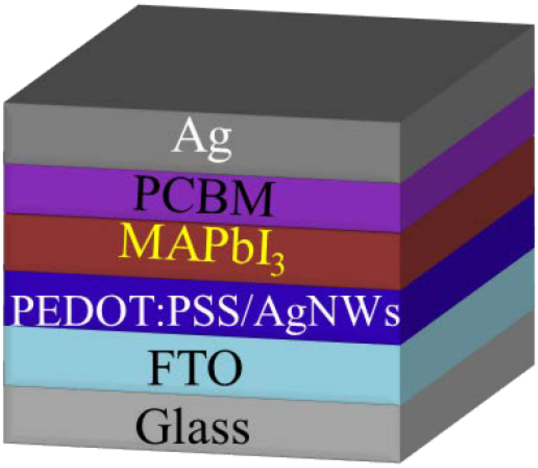

Figure S1 Schematic representation of structure of the cell device.

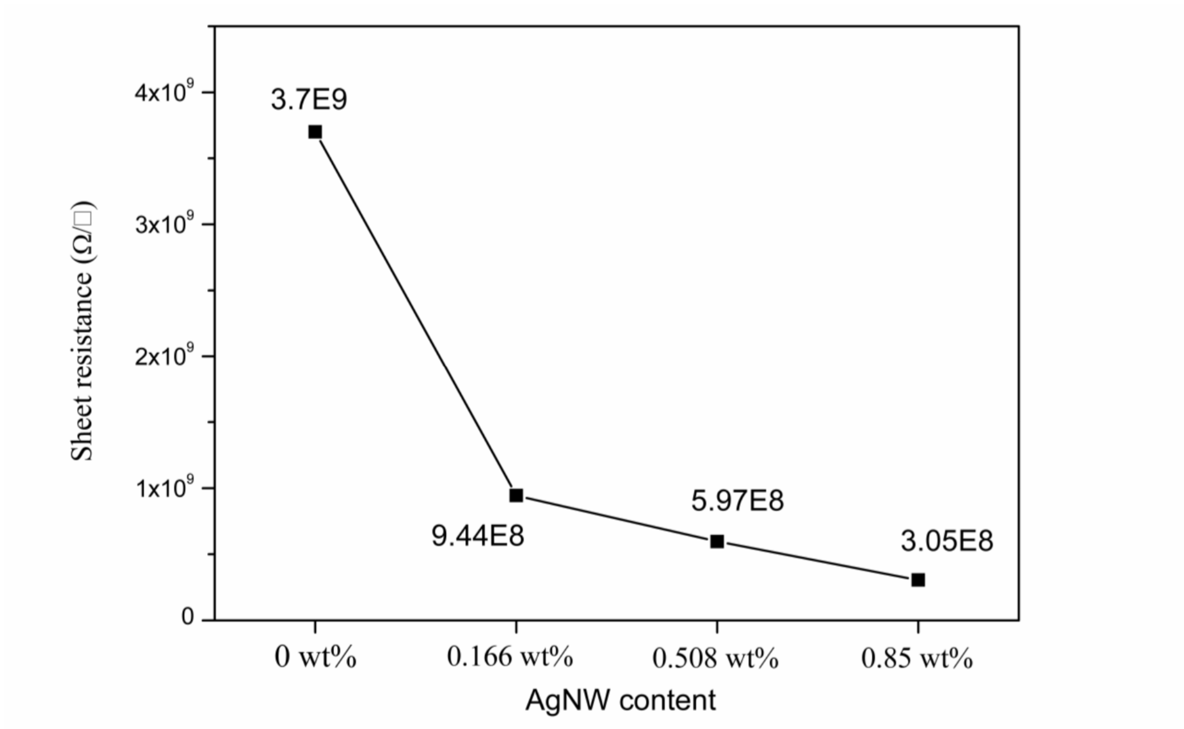

Figure S2 Variation on the sheet resistance of HTL with various AgNW contents.

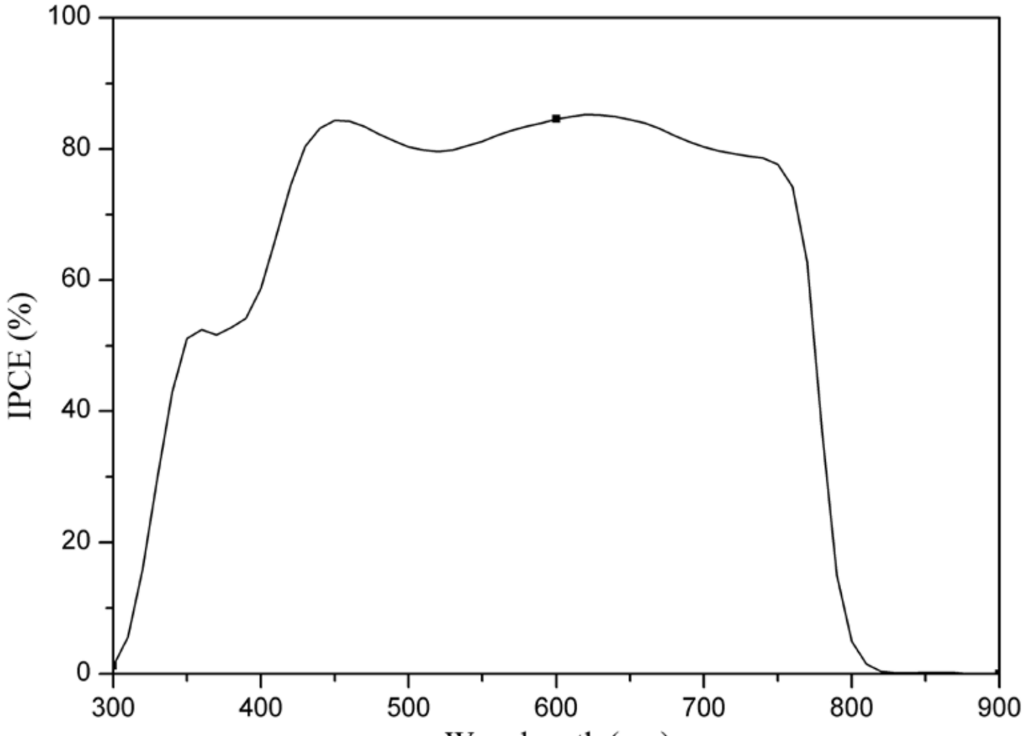

Figure S3 IPCE spectrum of PVSC-2.

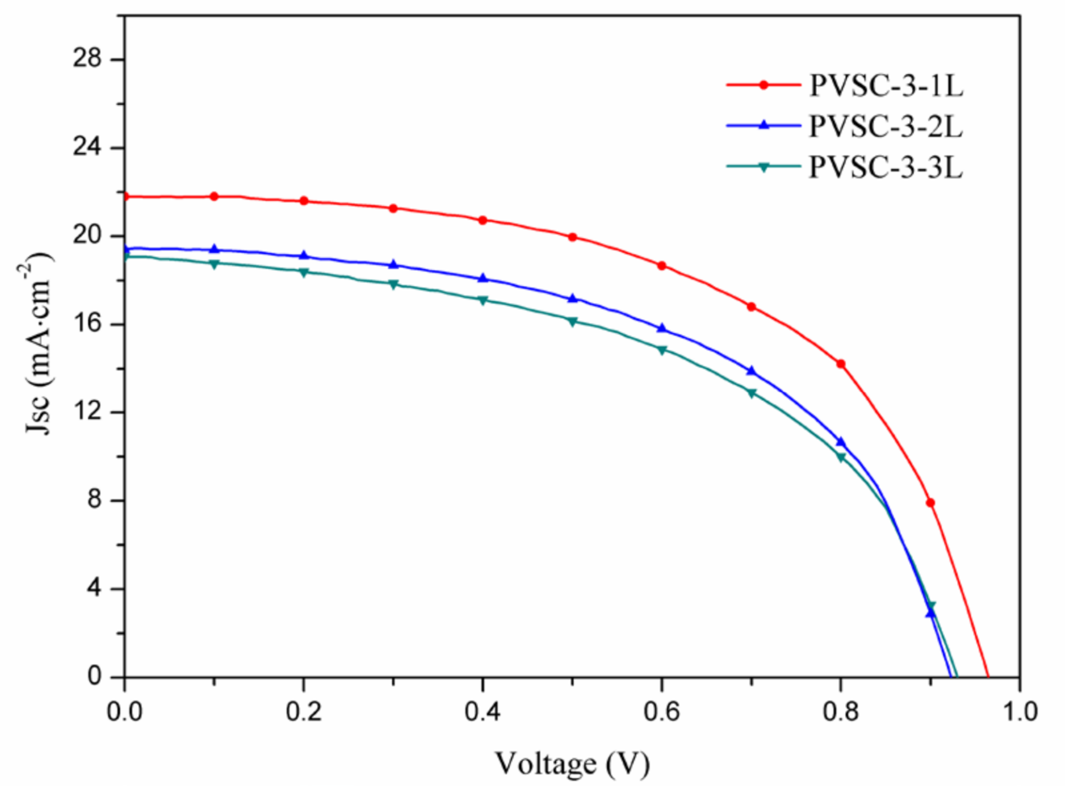

Figure S4 Photocurrent density-voltage curves of PVSCs with coating extra PEDOT:PSS layers.
